# Patient and citizen participation at the organizational level in health technology assessment: an exploratory study in five jurisdictions

**DOI:** 10.1017/S0266462323000417

**Published:** 2023-08-08

**Authors:** Hervé Nabarette, Marie-Hélène Chastenay, Jean-Claude K. Dupont, Isabelle Ganache, Ann N.V. Single

**Affiliations:** 1 Association Française contre les Myopathies – Téléthon, Evry, France; 2 Institut national d’excellence en santé et en services sociaux (INESSS), Montreal, QC, Canada; 3 Institut Pasteur, Legal Department, Ethics Unit and Centre de Recherche des Cordeliers, Sorbonne Université, Université Paris Cité, Inserm, Laboratoire ETREs, Paris, France; 4 Patient Voice Initiative, Sydney, NSW, Australia

**Keywords:** patient participation, technology assessment, health, public participation, organizational policy, organizational, case studies

## Abstract

**Objective:**

While patient participation in individual health technology assessments (HTAs) has been frequently described in the literature, patient and citizen participation at the organizational level is less described and may be less understood and practiced in HTA bodies. We aimed to better understand its use by describing current practice.

**Method:**

To elicit descriptive case studies and insights we conducted semi-structured interviews and open-ended questionnaires with HTA body staff and patients and citizens participating at the organizational level in Belgium, France, Quebec, Scotland, and Wales.

**Results:**

We identified examples of organizational participation in managerial aspects: governance, defining patient involvement processes, evaluation processes and methods, and capacity building. Mechanisms included consultation, collaboration, and membership of standing (permanent) groups. These were sometimes combined. Participants were usually from umbrella patient organizations and patient associations, as well as individual patients and citizens.

**Discussion:**

Although the concept, participation at the organizational level, is not well-established, we observed a trend toward growth in each jurisdiction. Some goals were shared for this participation, but HTA bodies focused more on instrumental goals, especially improving participation in HTAs, while patients and citizens were more likely to offer democratic and developmental goals beyond improving participation processes.

**Conclusion:**

Our findings provide rationales for organizational-level participation from the perspectives of HTA bodies and patients. The case studies provide insights into how to involve participants and who may be seen as legitimate participants. These findings may be useful to HTA bodies, the patient sector, and communities when devising an organizational-level participation framework.

## Introduction

Patient participation in individual health technology assessments (HTAs) has been frequently described in the literature in terms of goals and approaches. Additionally, a clear rationale for it has emerged especially concerning its role in adding insights and evidence to address gaps and uncertainties in the traditional evidence, including important local differences (1–4). However, individual HTAs represent only one possibility for participation. Gauvin et al.’s (5) public involvement in HTA mosaic describes two further levels, or “domains,” for participation: the policy level and the organizational level. Participation in defining policy, such as state obligations to cover healthcare services, is a consideration for policy makers. However, organizational-level participation is a matter for HTA bodies. Still it has been less described and may be less practiced than participation in individual HTAs. In this study, building on the work of Abelson et al. (6), Gauvin et al. (5), and Facey (4), we sought to better understand the use of patient participation at the organizational level by describing how patients currently participate in a sample of jurisdictions. In particular, we sought to create case studies describing it from both perspectives, patient or citizen participant and HTA body.

Gauvin et al. defined the organizational level as “the set of processes and procedures that affect the way that HTA agencies are directed, administered, or controlled,” including governance, prioritizing assessments, and commissioning research (5). In 2017, Facey’s mosaic further described areas for participation at the organizational level: developing and evaluating patient involvement processes and methodologies; influencing wider assessments methods, organizational processes, and values; and capacity building (4). Two recent examples in the literature of organizational level participation are the Canadian Agency for Drugs and Technologies in Health (CADTH) Patient and Community Advisory Committee (7) and the National Institute for Health and Care Excellence (NICE) “NICE Listens” deliberative public engagement programme which both seek public guidance, especially to address health inequalities (8).

Definitions for participation are numerous in the literature. Gauvin et al.’s mosaic for analyzing public involvement defines it with a seven-point scale to reflect increases in the amount of public control. However, they do not illustrate this scale with examples of organizational participation (5). Additionally, Facey characterized participation in HTA as ideally “dynamic,” “two-way,” and a “dialogue” (4).

Regarding whom to involve at the organizational level, Facey identifies roles for patients and patient associations in her mosaic (4). Gauvin et al. also include citizens and report that HTA bodies tend to seek patients and their representatives for individual HTAs, and people with a broader societal perspective for activities at the organizational level. This societal perspective is a key characteristic of citizens, who can be individuals, elected officials, or citizen group representatives rather than people with a specific patient, health professional, or scientific perspective. Patients can be defined as those with direct experience of a condition or technology, and patient associations as those who represent patients (5).

As with individual HTA involvement, the goals for organizational-level participation may be instrumental, democratic, scientific, or developmental (4;5), as described by Abelson et al. (6) and the Ontario Health Technology Advisory Committee Public Engagement Subcommittee (9). However, it may be particularly associated with democratic goals of making informed, transparent, and accountable HTA body decisions (5;6).

Furthermore, there is recognition that HTA is a value-laden process influenced by the perspectives of those involved. These perspectives influence not only the value determined for a health technology in individual HTAs (10), but the processes that set the rules for what is possible in those HTAs. These rules may be a barrier or enabler to the incorporation, in individual HTAs, of the value concerns and insights of patients (11) and patient-based evidence (12).

**Figure 1.**
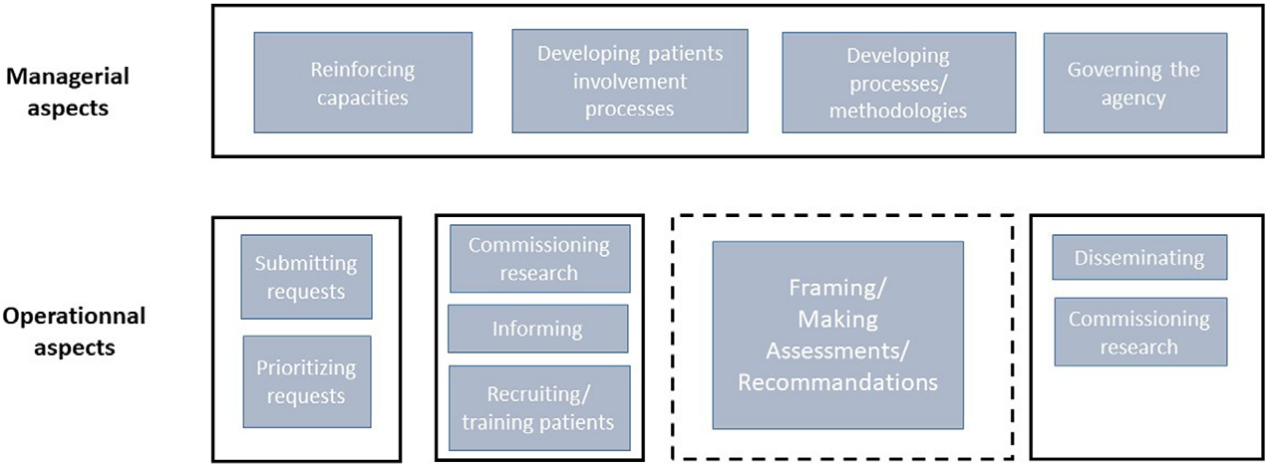
Organizational level in health technology assessment Patient participation at the organizational level: boxes with solid lines, managerial, and operational aspects (managerial: definition of rules, processes, strategic direction, and decisions; operational: organization linked to one or several assessments). Patient participation in an individual assessment: box with hatched lines.

In this study, we sought to better understand organizational level participation, especially for the managerial aspects (definition of rules, processes, strategic direction, and strategic decisions), distinguished from operational aspects (undertaking of one or many individual HTAs). For clarity, we developed a diagram to illustrate what was within the organizational level and what was outside the scope of our study (see Figure 1). Within this scope was participation in four areas:

1. governing the agency
2. developing HTA processes and methodologies
3. developing patient involvement processes, and
4. capacity building.

Case studies were developed from the perspectives of both the HTA bodies and the patient and citizen participants to describe:

1. Current practice in the four areas listed above
2. Who participates
3. How participation occurs
4. Insights about values, contributions, limitations, and how it should be further developed.

## Method

Due to the study’s descriptive and exploratory objectives, qualitative methods (13) were used to elicit case studies (14) and insights from HTA body and patient or citizen representatives with lived experience of organizational-level participation. As triangulating information from various sources is held as a standard in case studies, conjoint interviews and independent questionnaires were used, to cross-reference the perspectives of study participants while allowing them to express personal views or to highlight otherwise neglected aspects of the situation at hand (13).

### Recruitment

We used a pragmatic approach (15). The researchers contacted HTA bodies that provided diversity (large/small, new/longer established, medicine/nonmedicine) and that were already actively engaged in patient participation. Access was facilitated by the researchers’ professional affiliations with an HTA body or a patient association. This posture as researcher-practitioners improved their ability to understand context and ask clarifying questions. Interviewers and interviewees were from different jurisdictions.

Five agencies agreed to take part in the study: Belgian Health Care Knowledge Centre, KCE; Haute Autorité de Santé, HAS, France; Institut National d’Excellence en Santé et en Services Sociaux, INESSS, Quebec; Health Technology Wales, HTW; and Scottish Medicines Consortium, SMC. Additionally, CADTH agreed to take part in the questionnaire testing. One agency was contacted but declined.

Representatives from the HTA body and patients, citizens, or patient associations representatives with good knowledge and strong experience of organizational level participation in HTA were identified. The researchers worked with the HTA bodies to identify the patient and citizen participants (PCPs).

### Data Collection

A three-part questionnaire was developed to collect data in three stages:

1. *Context*: key background information about the HTA body and patient involvement in individual HTAs was completed by the interviewer using information on the HTA bodies Web sites and then validated by an HTA body participant prior to the Description interview.
2. *Description*: a joint HTA body/PCPs description of participation activities at the organizational level generated in a 90–120-min recorded Zoom interview with at least one HTA body representative and one PCP, covering the four areas of organizational level participation in the jurisdiction – including what, who, and how – and focusing primarily on one example which researchers agreed upon in advance with interviewees.
3. *Opinions*: study participants’ separate perspectives (HTA body, PCPs) about the value of this participation, contributions, limitations, and hopes for further development. A board member, director, or equivalent in the HTA body and patient association were also asked to contribute to this questionnaire. Study participants were encouraged to complete it collectively with others in their organization. Some completed it in online interviews and others in writing (see Table 1).

Questionnaires were tested with KCE and CADTH after receiving ethical approval (Research Ethics Committee of Greater Paris University Hospitals, CERAPHP Centre, PPOL study). The data collected in the CADTH pilot was not analyzed further, instead, the pilot was used to refine the method. It highlighted the need to reduce the time commitment for study participants, to focus on the managerial aspects of the organizational level, and on examples to elicit richer descriptions. Testing began in March 2020 and data collection was completed in September 2021.

**Table 1.** Summary of participating jurisdictions (2021)

	Belgium	France	Quebec	Scotland	Wales
Population (rounded to nearest 500,000)	11.5 million	68 million	8.5 million	5.5 million	3 million
Name of HTA body	Belgian Health Care Knowledge Centre (KCE)	Haute Autorité de santé (HAS)	Institut national d’Excellence en Santé et en Services Sociaux (INESSS)	Scottish Medicines Consortium (SMC) part of Health Improvement Scotland	Health Technology Wales (HTW)
Year established	2003	2005	2011	2001	2017
Recommendation status	Provides analyses of effectiveness, safety, and costs to payer	Advises Ministry on reimbursement and pricing	Advice mandatory for medicines Advice only for nonmedicines (Ministry)	Advises health boards/Area Drug and Therapeutics Committees	Advises National Health Service Wales (including local boards + trusts) to adopt or justify
Type of HTA	Medicines and medical services	Medicines and medical services	Medicines and medical services	Medicines (all new licensed and new indications)	Medical services
Other HTA bodies in the country	INAMI (National Health Insurance and Disability Institute) also evaluates certain drugs	National Cancer Institute – some cancer HTAs	Multiple actors at provincial and federal levels	Scottish Health Technologies Group, also part of Health Improvement Scotland	All Wales Therapeutics & Toxicology Centre
State-funded patient federations	Yes, 3 federations based on 3 languages	Yes, France Assos Santé (FAS)	No	No (SMC Public Involvement Network since 2015 + 3 umbrella organizations)	No
Patient/citizen individual HTA input	Variable and not systematized until 2021. Project team invites stakeholders (including patient representatives), often at a meeting at the beginning of project, and they or others may be consulted later	Online submissions since 2016 for health products + hearings and occasional working groups for devices. Public health interventions and medical procedures: frequent in scoping, working group + final consultation	Yes, expert advisory group membership, interviews, focus group, bespoke approaches	Structured submission since establishment + patient association representation at open meetings since 2015	Structured submission form since 2019 + bespoke approaches
Study participation Description (joint online interview)	1 HTA body rep 1 PCP	2 HTA body reps 3 PCPs	2 HTA body reps 2 PCPs	1 HTA body rep 2 PCPs	1 HTA body rep 1 PCP
Study participation Opinion	Online interview: 1 HTA body rep + 2 for post-interview validation 2 PCPs collectively +1 for post-interview validation	Online interview: 2 HTA body reps collectively 3 PCPs collectively	Online interview: 2 HTA body reps collectively + 1 for post-interview validation 2 PCPs collectively	Written questionnaire: 2 HTA body reps collectively 2 PCPs individually	Written questionnaire: 2 HTA body reps individually 2 PCPs individually

HTA, health technology assessment; INAMI, Institut National d’Assurance Maladie-Invalidité; PCP(s), patient and citizen participants; rep(s), representatives.

### Analysis

All interviews were transcribed. Relevant data were extracted and coded using a constant comparative method as proposed by tenants of grounded theory (16). This method was applied by shifting from comparison within cases – that is, between participants in the same jurisdiction – and between jurisdictions – that is, between cases comparison. The coding was made by a researcher (H.N.) and checked by two other researchers (M.-H.C., A.N.V.S.). To enhance credibility and reflexivity, the coding was discussed among researchers (H.N., M.-H.C., A.N.V.S.) and further refined until reaching an agreement, a process intended to make each researcher an independent judge of the qualitative material collected by his or her colleagues (17).

For the Description part, coding aimed to describe what, who, and how in the four areas. Concerning the how to question, we used:

- consulting, that is, “giving feedback” (18)
- collaborating, that is, “partnering in each aspect of the decision including the development of alternatives and the identification of the preferred solution” (18)
- and being a member of different standing (permanent) committees and boards.

We coded the type of PCPs according to the descriptions given by the study participants: patient or user; patient (user) association or umbrella organization representative; and citizen or member of the public.

For the Opinion part, we compared the themes in the HTA body responses to those of PCPs. These themes were primarily elicited from the participants’ direct speech but were also sometimes inferred from them, notably using Abelson’s goals for patient involvement (democratic, scientific, instrumental, and developmental). Themes were listed in order of frequency for HTA bodies versus for PCPs.

The tabulated data summaries were shared with study participants. Data were analyzed in French and English.

## Results

We will present our findings regarding how participation occurred and who participated, and summarize what we observed, before providing each case study and finally comparing the views of HTA bodies and PCPs. Table 1 summarizes the context of each jurisdiction and who took part.

As seen in Table 2, participation occurred by consultation, collaboration, and membership of a standing group – standing Patient and Public Involvement (PPI) group or standing HTA committee – or board. It primarily involved four types of participants:

1. representatives of patient umbrella organizations,
2. representatives of patient associations,
3. individuals identifying as citizens (including public members),
4. individuals identifying as patients.

A trend toward increased participation is visible with the recent creation of PPI groups, patient membership on standing committees, and significant consultations or collaborations.

**Table 2.** Mechanisms and types of patients at the organizational level

Mechanism	Organizational level role	People representing patient/citizen perspective	Evolution
Membership of board (KCE, HAS, INESSS)	Board: strategic decisions, internal functioning (e.g., audit). Major role concerning governing the HTA body.	1 patient (HAS + INESSS) or 2 patients (KCE) on board. KCE board also includes a member of the House of Representatives	Initially no patients on boards, but added in response to advocacy for role (KCE 2015, HAS 2017, INESSS 2020)
Membership of standing PPI group (HAS, SMC, HTW)	Standing PPI group: decide (HTW) or advise (HAS, SMC) on patient involvement processes, sometimes evaluation processes and methodologies, or capacity building	At least 50% of patients/citizens: representatives of patient umbrella organization (SMC) or public representatives from standing HTA committee (HTW, SMC), or individual patients (HAS)	Set up after establishment of HTA body (HTW 2019, HAS 2020), set up at establishment and later revised and strengthened (SMC 2014)
Membership of standing HTA committees (HAS, INESSS, SMC, HTW)	Standing HTA committee: participate in review and validation of HTA processes and methodologies (in addition to operational role in individual HTAs)	2 (INESSS, HTW) or 3 (HAS, SMC) patients or citizens recruited by open advertisement for each standing HTA committee	Added after standing HTA committees established (HAS 2015, HTW 2019, INESSS 2011–2016–2018) and at establishment in 1 HTA body (SMC 2002), where number of citizens (“public partners”) has grown
Consultation or collaboration with umbrella organizations (KCE, HAS, SMC), or patient associations (SMC, HTW, HAS, KCE)	Project-specific roles including defining and developing patient and public involvement processes; defining new HTA pathways and developing existing HTA processes; scoping and developing capacity building	Representatives of patient associations and umbrella organizations only	SMC initially consulted individually with patient associations before creating a public involvement network and registering associations as “patient group partners”
Consultation or collaboration with individuals (KCE, INESSS)	Project-specific role including: contributing to the definition of patient involvement processes, or to strategic plan	Workshops with individual patients and association representatives, ad hoc groups with citizens on standing HTA committees and individual patients	

*Note:* This table shows the different mechanisms used, their role, and the type of participants.

HAS, Haute Autorité de santé; HTA, health technology assessment; HTW, Health Technology Wales; INESSS, Institut National d’Excellence en Santé et en Services Sociaux; KCE, Belgian Health Care Knowledge Centre; SMC, Scottish Medicines Consortium.

In the case studies, there were examples of the four areas of participation at the organizational level. In the area of governance, patient board membership was the primary mechanism of participation. However, consultation with patient associations, umbrella organizations, individual patients, and citizens was also reported on strategic projects.

In patient involvement processes, participation took the form of consultation and collaboration to define, review or develop the processes with all four types of participants observed. This participation was sometimes facilitated through a standing PPI group.

In HTA processes and methods, consultation was the primary mechanism of participation, however, patients or citizens of standing HTA committees also played a role in developing processes.

Capacity building was another area for participation with standing PPI groups identifying training needs and developing training programs. Additionally, umbrella organizations or patient associations facilitated training by inviting HTA bodies to speak to their staff and association members.

### Case 1: KCE, Belgium – With an Example of a Patient Involvement Process Project

In Belgium, representatives from two umbrella patient organizations (one for French speakers and one for Dutch speakers) have been on the board of the HTA body, KCE, since 2015 following a decision by the Minister of Health aimed at strengthening patient representation (see Table 2). Umbrella associations were also granted government funding in line with this aim. A member of the House of Representatives and a representative of sickness funds run by users also sit on this board. Additionally, KCE can organize consultations and collaborations with umbrella organizations, sickness funds, patient associations, or individual patients.

In 2019, KCE adopted a patient involvement policy for individual HTAs which was developed without consulting patients (19). However, in 2020–21, KCE collaborated with various patients and citizens to determine how patients would be involved in individual HTAs. Different workshops were organized, according to the language (French or Dutch) and the type of patient (umbrella/ sickness fund/ patients and association representatives). Some workshops were online because of the COVID-19 pandemic. A pilot was also undertaken with the patient association for mental health, Psytoyens. An outcome of this unfolding initiative was that KCE and the umbrella organizations decided to meet annually to identify the projects which must integrate patient involvement, and the relevant patient associations (20).

### Case 2: HAS, France – With an Example of an HTA Process and Method Project

A patient is on the board of HAS, but this position is not provided for by the Law. In addition to patient members on the standing HTA committees, there is an advisory standing PPI group (see Table 2). Consultation or collaboration with umbrella organizations or patient associations is frequent. The umbrella organization, France Assos Santé, was established by legislation and is funded by the State in line with a policy of “health democracy.” It is composed of 85 accredited user associations.

In 2021, HAS defined new modalities for early access to medicines. The umbrella organization and patient associations collaborated with HAS in defining patient involvement in this new process. This included opportunities to attend hearings, access to the industry file describing the therapeutic use protocol and planned data collection, and coconstruction of the questionnaire to gather the opinions of patient associations. The patients on the standing HTA committee also played an important role in defining the general early access mechanism, that is, increased use of PROMs in the data collection that will accompany the early access period, and, critically, the definition of the criteria for a drug to be eligible for early access. Patient associations were also consulted on the general mechanism.

### Case 3: INESSS, Quebec – With an Example of a Participation in Governing the HTA Body Project

In Quebec, organizational participation relies on a patient on the board of INESSS and consultations with individual patients or citizens (see Table 2). Consultation with patient associations is not frequent. Quebec has developed its own distinct partnership model for patients in the health and social service system that is based on the figure of the expert patient. It is used by INESSS, which relies in part on people trained in this model: for example, a patient board member and a patient coach coordinator who have links with the Université de Montréal-supported Centre d’Excellence sur le Partenariat avec les Patients et le Public (CEPPP). The patient coach coordinator plays a pivotal role in patient engagement planning, patient recruitment, and coaching (operational aspects).

Citizen members on standing HTA committees and individual patients experienced in participating in INESSS committees and working groups were consulted about the 2021–24 INESSS strategic plan via online group discussions. INESSS’s patient coach coordinator also participated in this reflection and cofacilitated the sessions. The consultation was a response to an objective in the previous strategic plan regarding structuring the contribution of patients and citizens for a stronger integration of the patient perspective in INESSS work.

### Case 4: SMC, Scotland – With an Example of a Standing PPI Group

In Scotland, the organizational participation relies on an advisory standing PPI group, citizens on the standing HTA committee and consultations with patient associations (see Table 2). In 2014, following independent reviews of SMC and patient associations’ requests for partnership, SMC enabled patient associations to register as “patient group partners” in its Public Involvement Network (PIN), a formal network for consultation and training. It also created the PIN Advisory Group, a standing group that includes representatives from three umbrella organizations (Scottish Cancer Coalition, Genetic Alliance UK, and The Alliance), and three citizen members of the standing HTA committee. The PIN Advisory Group evaluates and develops patient involvement processes, including developing support tools and training. It has helped to implement recommendations from the independent reviews, such as participating in process and method developments, which go beyond their initial remit. For example, the PIN Advisory Group contributed to creating an alternative evaluation pathway and a validation process for ultra-orphan drugs. On this occasion, SMC also consulted the patient associations of the PIN.

### Case 5: HTW, Wales – With an Example of a Standing PPI Group

In Wales, organizational participation usually occurs through the decisional standing PPI group and consultations of patient associations (see Table 2). HTW was formed after HTA bodies in England and Scotland. It may draw on patient expertise from the wider United Kingdom.

The Patient and Public Involvement Standing Group (PPISG) comprises public members of the standing HTA committee, called Public Partners, and an equal number of PPI advisors, including international experts. PPISG determines processes for patient participation and using patient-based evidence (such as primary research and published literature) in each HTA. It has influenced the HTA processes and methodologies by advocating for patient evidence to be treated the same as clinical and economic evidence, including how it is documented, and introducing protected time for dialogue about patient aspects at standing committee meetings. In addition, it guides capacity building by defining webinar contents for patient associations in collaboration with HTW leaders.

### Opinions Compared

Concerning the opinion questionnaire, HTA bodies and patient and public participants consistently acknowledged the value/contribution associated with participation at the organizational level (see Table 3). A common theme was the democratic goal of “legitimacy” under two complementary expectations: of participation (of patients directly affected by HTA recommendations), and of transparency and accountability (toward broader public). Another common theme was the scientific goal of improving HTA by integrating the specific perspective of patients and their associations. HTA bodies and PCPs sometimes hold distinct rationales. For example, the HTA bodies all stressed the link to improving individual HTA assessments. PCPs sometimes point out limitations, such as being in numerical inferiority in standing HTA committees or on the board, or the use of technical language.

**Table 3.** Study participants’ perspectives on the value (+) of participation at the organizational level and limitations (−) to its use (from most common to least common)

HTA body	Patient and citizen participants (PCPs)
*(+) Improving the PPI processes (all)* France: “It is logical to organize the mode of contribution with the users since the HAS asks them for contributions” Scotland: “Partnership working has strengthened our engagement processes, which has in turn led to an increase in participation. It has also significantly increased the satisfaction of patient groups participating in HTA” *(+) Legitimacy, transparency, national policy (Belgium, Scotland, Wales)* Scotland: “Participation is a national policy and organizational imperative” Wales: “Public care systems are directly funded by citizens and thus should, I believe, reflect their priorities. This cannot happen unless there is public and patient participation at the organizational level in the HTA body” *(+) Specificity of patient perspective (Belgium, Quebec, Wales)* Belgium: “Contributes to the human side of the work, even at the organizational level” Quebec “In working with them, they will act as a kind of lighthouse, indicating what to aim for, the direction to follow” *(+) Internal processes improvement (Wales)* Wales: “Shaping how organization works, improvements to process” *(+) Reach out to the public (Wales)* Wales: “Organizational participation can also help HTA bodies reach out to the public” *(−) Conflict of interest (Quebec)* Quebec: “Due to possible conflicts of interest, especially when they have an advocacy role outside the HTA agency, the role of patient groups must be carefully reflected on, the conflicts are identified, assessed and managed” *(−) Resources (especially for operational aspects) (Belgium, Quebec)* Belgium: “These processes require resources: time, staff, energy” *(−) Political and professional structure (Wales)* Wales: “Limitations only come from political and professional structure”	*(+) Legitimacy, democracy, trust, transparency, fairness (Belgium, France, Scotland, Wales)* Belgium: “Patients and citizens are the primary stakeholders in the studies, so it makes sense for them to be involved at different levels” France: “It is the legal mission of our umbrella organization to give advice to public authorities on health policy, and therefore for us also on the health product chain” *(+) Specificity of patient perspective, decision enrichment, humanity, diversity (Belgium, France, Quebec)* Quebec: “Patients bring humanity, appropriateness, and diversity” *(+) Improving the PPI processes (Wales)* Wales: “Ensure that the ‘public voice’ is heard in the assessment and appraisal panel meetings” *(+) Sense of purpose (France)* France: “Feeling of being useful to society and of contributing back after having benefited from a care pathway paid for by the state (and taxpayers/society)” *(−) Proportion of patients in committees (France, Quebec)* France: “We are only 2 user members out of 22 in the Committee, we must dare to express ourselves” *(−) Technical language (Belgium, Quebec)* Quebec: “The HTA body translates in its language patient experiences whereas it would be better to reach a speech easy to understand by all in a strategic planning” *(−) Clarity of the processes (Belgium)* Belgium: “Understand at what point in the development of a project, a study programme, a selection process, our federation and/or a patient organization can participate, as there was no clear process until now” *(−) Linguistic issues (Belgium)* Belgium: “The linguistic requirements of some meetings may limit the patient profile that could participate” *(−) Patients’ profile (Quebec)* Quebec: “The HTA body is inclined to choose people who resemble it, with the danger that they become ‘professionals’” *(−) Perception of expertise (Scotland)* Scotland: “We can sometimes be seen as not on the same level as clinicians or health economists in HTA”

*Note:* Responses from participants when asked about value, contribution, and limitations of participation at the organizational level.

HAS, Haute Autorité de Santé; HTA, health technology assessment.

Opinions varied between HTA bodies and PCPs on the desired evolution of this involvement (see Table 4). HTA bodies often expressed a desire to: increase the role of patients in the conduct of assessments (operational involvement); implement the procedures defined with patients; and capitalize on existing assets for patient involvement. PCPs often cited: bringing patients closer to the HTA body decision makers; reinforcing the principle of organizational participation (in terms of how much occurs, legal status); the necessity for the HTA body to speak more directly to the population (not just government and industry) and to develop its relationship with patient associations; an expanded role for patients in HTAs (including participation in horizon scanning); and increasing their training and experience (e.g., in relation to access to emerging drugs).

**Table 4.** Study participants’ perspectives on the desirable evolution for participation at the organizational level (from most common to least common)

HTA body	Patient and citizen participants (PCPs)
*Increase patient role in implementing specific HTAs (e.g., coproduction, submission, prioritization, impact study) (Belgium, Quebec, Wales)* Quebec: “It would also be meaningful to make it possible for an input in quality control and prioritising” *Implementing the patient engagement procedures recently defined with patients (Belgium, France)* France: “A roadmap on strengthening user engagement in HTA has been co-constructed, we need to follow up its implementation with users” *Capitalize on existing assets (e.g., patients on committees, on board, link with university…) (France, Quebec)* Quebec: “We must capitalise on what was already done by citizens on committees and on the board of trustees, and increase the links with university (especially Centre d’Excellence sur le Partenariat avec les Patients et le Public, CEPPP) and other citizen participation bodies” *Evaluate collaborations (Belgium, France)* Belgium: “Experiment with several projects and then re-evaluate” *Increase place in governance (executive) (Scotland, Wales)* Scotland: “There could be stronger connection between PIN Advisory Group and SMC Executive” *Increase dedicated budget (Scotland, Wales)* Wales: “We have focused mainly on mainstreaming robust PPI into our evidence processes, we must grow budget for involvement in all aspects, especially topic submission, audit on the adoption of HTW national guidance” *Understand impact (Scotland)* Scotland: “We need to understand the impact of this involvement on decision making” *Patient representation within the organization (Wales)* Wales: “There could be a broader representation across organization”	*Bring patients closer to the governance of the HTA body (Quebec, Scotland, Wales)* Scotland: “Greater engagement between PIN advisory group and executive” Quebec: “Adopt tools and training to facilitate patient/HTA body discussion management” *Reinforce the participation principle at the organizational level (e.g., principle at the national level, at the level of the HTA body, regulatory status of patient on board) (Belgium, France, Quebec)* France: “Consideration should be given to the legal representation of user associations on the HAS board” Quebec: “Increasing the share of organizational participation in various public bodies” *HTA body must speak more directly to the population/not just government and industry (Belgium, Quebec)* Belgium: “Communicate more studies to the public, in an accessible language” *Developing relationship with patient associations (Belgium, Quebec)* Quebec: “The voice of associations and activists should be taken into account more. The agency relies on individuals not groups, it is the patient partnership model, but the patient experts and the patient partner model are only one form of the citizen voice” *Increase patient role in HTAs, including participation in horizon scanning, prospective works (Quebec, Scotland)* Quebec: “Value of making greater use of the following operating modes: promoting HTA issues from citizens; moving from consultation to co-production; participate to prospective medicine of the future” *Patient role: continue gaining experience (e.g., board participation, access to drugs that are emerging) (Belgium, Scotland)* Scotland: “Have many treatments which come for appraisal therefore we have the expertise to be able to contribute to the wider access policy environment” *Evolution (Wales)* Wales: “Expectation that PPI will evolve rather than stand still”

*Note:* Responses from participants when asked how they would like to see patient participation developed further.

CEPPP, Centre d’Excellence sur le Partenariat avec les Patients et le Public; HAS, Haute Autorité de Santé; HTA, health technology assessment; PIN, public involvement network; PPI, patient and public involvement; SMC, Scottish Medicines Consortium.

## Discussion

In contrast to individual HTAs, there is little literature or guidance on patient and citizen participation at the organizational level in HTA. We observed that this participation – as a distinct concept that requires clear strategy, goals, and approaches – was not well-established in the minds of people working in HTA bodies and PCPs. This confusion resulted in some study participants at times contributing data about individual HTA participation which was later omitted from the study.

### Areas of Participation

While participation was evident in the four areas of study focus, the development of patient involvement processes was the most frequent with clear rationales that were articulated and new and improved processes implemented. However, developing HTA processes and methods were also observed, for example with early access in France or ultra-orphan medicines in Scotland.

We observed that organizational participation tends to increase in HTA bodies once implemented. For example, SMC’s PPI standing group has exceeded its original remit. This is in line with patient demand, but also supported by HTA body testimony that past collaborations and continuity of relationships are enablers of further organizational participation.

In terms of participation in developing HTA processes and methodologies, we did not see a systematic consultation principle, such as the fixed plan for consultation in the construction of the European HTA system (21).

### How Participation Occurs

In our cases, participation was observed to primarily occur through membership of a standing group, such as a board or PPI group, but could also occur through consultation or collaboration on a specific issue. These mechanisms suggest that three of Gauvin et al.’s grades of involvement are of particular relevance to participation in managerial aspects: commenting (taking input but with no obligation to respond such as the Quebec example); collaborating (providing guidance or advice such as the examples from Belgium, France, and Scotland); and engaging (a joint group with decision-making authority such as the Wales example). The last one implies a very high level of influence that could be associated with the PPI needs of medical services HTA. However, these categories, based on the degree of control the public can exert, may be a blunt tool for understanding mechanisms if not considered within the context, including the rationales and aims for participation (9), who takes part and the presence of enablers or barriers (22).

Additionally, we observed that consultation and collaboration are sometimes combined (Belgium, France, Scotland), possibly also with an additional role played by patients or citizens on standing HTA committees (France). This can be preceded by patient participation upstream with the government (France), that is, participation in the policy domain in Gauvin’s typology (5).

### Who Participates

We found individuals, patient associations, and umbrella organizations participating. This variety is likely to stem from participation experience in individual HTAs. Our study suggests that, where present, patient federations are particularly sought for organizational-level participation. This may be because they have resources and are viewed as legitimate as they represent a diversity of patient associations and may be legislated entities in support of health democracy policies (France, Belgium). In the absence of a federation, the SMC created a formal network of patient associations for consultation. However, some HTA bodies also work with individuals. These might be patients regarded as legitimate due to their training as expert patients (Quebec) or citizens with strong networks and experience in patient communities (Wales), or an interest in improving patient experiences and reducing inequalities (Scotland). CADTH’s interest in inequities resulted in the creation of a patient and community group comprising individuals with a diversity of lived experiences rather than representatives of organizations or viewpoints (7). The different contexts and goals influence these variations between jurisdictions (6). Moreover, there are debates on the legitimacy of individual patient and citizen participants as shown by the testimonies of PCPs in Québec who suggested working more with patient associations to complement the expert patient model.

### Why Participation Occurs

We did not see a general framework addressing the potential breadth of organizational-level participation. Patient and citizen participation can be the result of different political objectives, such as enacting participation policies, responding to patients’ requests to review how HTA is done, or deciding to appoint patient members to board. However, study participants also expressed clear rationales for this participation. Although there were arguments in both groups in support of the democratic and scientific goals of patient participation, three differences emerged (see Tables 3 and 4). The HTA bodies were more likely to insist on instrumental goals, for example, making PPI processes work better. The PCPs were motivated by developmental goals, for example, capacity building to influence access to medicines. PCPs’ democratic goals included: increasing the proximity of patients and citizens to HTA body governance, progressing toward a mandatory principle of participation, and orientating the HTA body more toward the public in addition to government and industry.

### Limitations

Our study includes HTA bodies that have agreed to participate and be transparent on this subject and therefore were more likely to have positive views on the topic. We did not study participation in the operational aspects, but it may have a major role, as in the case of topic proposals submitted by patients to HTA bodies. Additionally, we did not study how the type of technologies (medicines or medical services) assessed or the size of the organization might influence the approach to organizational participation. Further work could more systematically compare participants’ roles and evaluate the influence of participation and associated factors.

## Conclusion

Our findings add to the work of Gauvin et al. (5) and Facey (4) to provide clear rationales for organizational-level participation from the perspectives of HTA bodies and PCPs, especially concerning managerial aspects. The five case studies provide insights into how to involve participants as well as who may be seen as legitimate participants. These findings may be useful to HTA bodies, the patient sector, and communities who seek to devise a framework for organizational-level participation.
